# Dislocation‐Driven Formation of Oriented Macroperiodic Metastructures of Curved Single Crystal Lattices in Glass

**DOI:** 10.1002/advs.202412833

**Published:** 2025-02-04

**Authors:** Evan J. Musterman, Volkmar Dierolf, Himanshu Jain

**Affiliations:** ^1^ Materials Science and Engineering Department Lehigh University Bethlehem PA 18015 USA; ^2^ National Synchrotron Light Source II Brookhaven National Laboratory Upton NY 11973 USA; ^3^ Physics Department Lehigh University Bethlehem PA 18015 USA

**Keywords:** chalcogenide, crystal, dislocation, glass, laser

## Abstract

Single crystals fabricated in glass by localized heating can develop uniquely deformed lattices stabilized by the surrounding amorphous medium. The development of lattice curvature appears to be intrinsic to the crystal growth process in some systems, while the result of the locally changing crystallography in others. In this work, a model laser‐fabricated rotating lattice Sb_2_S_3_ crystal grown in stoichiometric glass is used to demonstrate fabrication of novel macroperiodic metastructures that utilize intrinsic lattice curvature superimposed with subtle crystallographic influences. The limited availability of slip systems drives the lattice curvature magnitude to vary with crystal growth direction, maximizing for lattices aligned with the predominant 1/2[100] Burgers vector along with corresponding increases in dislocation density. Misaligned lattice orientations form smaller secondary lattice curvatures arising from misaligned Burgers vectors with further elastic contributions. Over extended crystal growth, these secondary components align the lattice to rotate about either the <001> or <010> crystal axes forming repeating metastructures of lattice orientation with periodicity 20–160 microns in length. The mechanistic approach used in this work may be expanded to other systems with known slip systems to better understand and design macroperiodic metastructures.

## Graphical abstract

The anisotropic nature of lattice deformations is shown to produce novel macroperiodic lattices with a periodicity of 10s of µm in model Sb_2_S_3_ single crystals laser‐fabricated in glass. While relieving crystallization stresses, the limited availability of slip systems imparts a crystallographic dependence on the lattice curvature to align the rotation axis over extended crystal growth and create the observed metastructures.

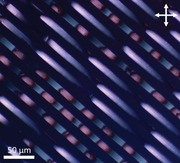

## 1 Introduction

Crystal lattices grown under confinement, like that provided by a surrounding amorphous medium, can deform during growth to develop lattice curvature and strain within otherwise single crystal grains.^[^ ^1^, ^2^ ^]^These types of uniquely deformed lattices can lead to new local lattice environments and lower symmetries for emergent,^[^ ^3^, ^4^, ^5^ ^]^ enhanced,^[^ ^6^, ^7^ ^]^ or tuned^[^ ^8^, ^9^, ^10^ ^]^ properties. Initial observations in glassy materials demonstrated uncontrolled lattice curvatures occurring across several randomly orientated grains^[^ ^11^, ^12^ ^]^ limiting technical applications larger than those individual grains. However, single crystals fabricated in glass by localized heating^[^ ^13^, ^14^ ^]^ have demonstrated similar, but controllable, lattice curvatures over indefinite length scales (µm to mm or beyond). Controllable lattice curvatures observed thus far can be broadly categorized as rotating,^[^ ^15^ ^]^ spiral,^[^ ^16^, ^17^ ^]^ graded,^[^ ^7^, ^18^ ^]^ or bending lattices,^[^ ^1^ ^]^ which can arise plastically from unpaired dislocations^[^ ^19^ ^]^ or elastically from strain gradients.^[^ ^20^ ^]^ Many of these crystals have low symmetries and limited slip systems^[^ ^1^ ^]^ raising fundamental questions regarding relative plastic and elastic contributions and whether lattice curvature is intrinsic to the fabrication process, influenced by the continuously changing local crystallography, or some combination thereof.

Understanding the anisotropic nature of deformation mechanisms is applicable to many functional materials^[^ ^21^, ^22^, ^23^ ^]^ including all types of curved crystal lattices formed in glass. Those crystals with a strong preferred crystallographic growth direction will bend their lattices to match the growth direction imposed by laser scanning.^[^ ^1^, ^24^, ^25^ ^]^ These bent lattices are formed by changing laser scanning directions and from misoriented crystal seeds, and extend only insofar as there is misalignment between the two growth directions before vanishing. This bending lattice curvature is apparently intrinsic to the misorientation of the lattice and not the crystal growth process itself. In contrast, Veenhuizen et al.^[^ ^18^ ^]^ showed this same vanishing bending lattice behavior in LiNbO_3_, which prefers [0001] axis crystal growth superimposed with a radially symmetric lattice curvature forming a graded lattice across the transverse crystal cross section. Despite the disappearing bending lattice curvature, the graded lattice continues indefinitely, demonstrating that graded lattice curvature is intrinsic to crystal growth for a constant lattice orientation along the crystal length. Komatsu and Honma^[^ ^16^ ^]^ showed a similarly indefinite lattice curvature for spiral growth of β’Gd_2_(MoO_4_)_3_, but with a continuously changing lattice orientation which spirals about a constant preferred [110] crystal growth direction. Of the known examples of lattice curvature, rotating lattices have the fewest restrictions during crystal growth where both the lattice orientation and crystal growth direction change continuously along the crystal length.^[^ ^1^, ^15^ ^]^ These dynamics superimposed with a limited availability of slip systems yield the greatest opportunity to elucidate subtle crystallographic tendencies from lattice deformations intrinsic to the crystal growth process. Such effects become further compounded when crystal growth is extended to practical lengths scales (∼ mm or longer) and the lattice may evolve through all possible orientations.

In this work, we establish the crystallographic and intrinsic nature of lattice curvature in the model Sb_2_S_3_ crystals laser‐fabricated in glass,^[^ ^15^ ^]^ ultimately leading to the formation of a new type of metastructure of repeating lattice orientation 10s of microns in length. Already as a functional material, Sb_2_S_3_ has garnered interest as a phase change material,^[^ ^26^ ^]^ photocatalyst,^[^ ^27^ ^]^ battery anode,^[^ ^28^ ^]^ and solar cell^[^ ^29^, ^30^ ^]^ where efficiency and stability gains have been demonstrated by cation doping and subsequent lattice distortions.^[^ ^31^ ^]^ As a model system, Sb_2_S_3_ is well‐studied for laser crystallization^[^ ^15^ ^]^ with a mechanistic understanding of lattice curvature through direct observation of unpaired edge dislocations^[^ ^32^ ^]^ over a subset of orientations. Extrapolating the dislocation‐driven lattice rotation observed in Sb_2_S_3_ – which is highly anisotropic and has a limited availability of slip systems – to all possible lattice orientations raises fundamental questions about the effects of misaligned Burgers vectors, relative dislocation densities, and potential elastic contributions toward lattice curvature. These questions of anisotropic single crystal plasticity^[^ ^21^, ^22^, ^23^ ^]^ can be surmised for laser‐fabricated Sb_2_S_3_ crystals as: *does the lattice curvature continue unperturbed as the crystal is grown indefinitely and can it be useful for functional applications?*

## 2 Results and Discussion

### 2.1 Initial Crystal Growth

#### 2.1.1 Growth Direction Dependence of Lattice Curvature

**Figure 1:**
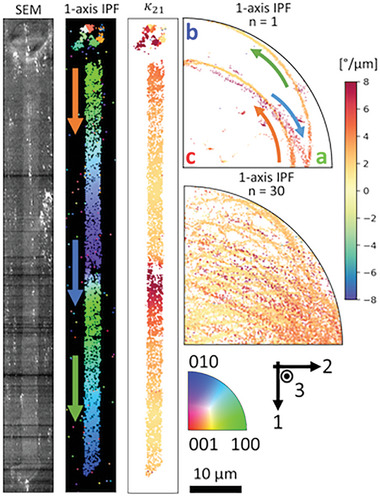
Local orientation and lattice curvature analysis by electron backscatter diffraction (EBSD) of Sb_2_S_3_ crystal line fabricated in stoichiometric glass with 3.25 mW laser power scanned at 100 µm s^−1^. From left to right: scanning electron microscope (SEM) image, 1‐axis IPF colored map, local κ_21_ map, 1‐axis IPF colored with local κ_21_ component. Arrows indicate the correlation of orientation in the IPF to the spatial regions in the map. Cartesian coordinate system defined by 1‐, and 3‐axes as the laser scanning and propagation directions, respectively, and the 2‐axis orthogonal to the 1‐3 plane. Below the individual 1‐axis IPF is an aggregate 1‐axis IPF for 30 independently fabricated crystal lines under identical laser parameters and half the spatial resolution for EBSD scans.

To better understand any subtle crystallographic dependence of lattice curvature, previous measurement methods,^[^ ^15^, ^32^ ^]^ were refined to characterize the locally changing lattice curvature of individual crystal lines as they rotate continuously through a subset of lattice orientations and growth directions. **Figure** **1** shows a typical Sb_2_S_3_ rotating lattice crystal line^[^ ^15^ ^]^ grown in stoichiometric glass with 3.25 mW laser power scanned at 100 µm s^−1^ oriented such that the 1‐axis is the laser scanning direction, the 3‐axis is the laser propagation direction, and the 2‐axis is orthogonal to the 1‐3 plane.^[^ ^1^ ^]^ Without considering the polycrystalline seed near the top of the map,^[^ ^33^ ^]^ the crystal starts growing nearly parallel to the *a*‐axis, the <100> direction. This is shown in Figure 1 as a green color just below the seed in the top 1‐axis inverse pole figure (IPF) colored map and by the position of the data points (individual map pixels) in the top 1‐axis IPF unit triangle. As the crystal continues to grow, the predominant κ_21_ lattice curvature component, defined according to Equation (1) in Experimental Section, rotates the lattice orientation and growth direction about the 2‐axis continuously nearer the <010> direction then back with some misalignment. The lattice rotation appears as a gradual change in color within the 1axis IPF map and as a continuous line of data points in the top 1‐axis IPF. The local κ_21_ lattice curvature component determined from this orientation data is plotted in Figure 1 as a map and indicated by the color in the top 1‐axis IPF. Along this single crystal line, κ_21_ changes by nearly 8° µm^−1^, but from this limited example, the cause of this change is ambiguous between a spatial dependence from the location along the length of the crystal line or a crystallographic dependence from the local lattice orientation. This ambiguity is partially resolved by focusing on the top 1‐axis IPF where larger κ_21_ occurs when the crystal grows toward the <100> axis.

By growing many crystal lines from randomly (or nearly randomly)^[^ ^34^ ^]^ oriented crystal seeds, any spatial correlations can be mitigated relative to crystallographic effects and a greater subset of lattice orientations can be measured. Following this logic, the electron backscatter diffraction (EBSD) analysis in Figure 1 was repeated for 30 crystal lines independently nucleated and grown under identical laser parameters in the same glass. The aggregated lattice orientations and κ_21_ local lattice curvatures are plotted in the bottom 1‐axis IPF in Figure 1 – see Figure S1 (Supporting Information) for other stereographic projections. For all crystal lines, κ_21_ was the predominant lattice curvature component, which increased when growing toward and through the <100> crystal axis following the same trend as the single crystal line. These relative changes in κ_21_magnitude with local lattice orientation indicate a crystallographic dependence to lattice curvature, which despite the wide variation, never truly reaches zero for any lattice orientation, also indicating κ_21_ is intrinsic to this fabrication method. Furthermore, the relative changes in κ_21_ can be considered with respect to Equation (2) in Experimental Section, where κ_21_ = *b*_1_ρ_2_ in the absence of elastic contributions. For a constant dislocation density, ρ_2_, lattice curvature will only depend on the projection of the Burgers vector along the crystal growth direction. This follows that κ_21_ is then maximized when the largest Burgers vector, the 1/2[100], is parallel to the growth direction – following previous observations over a smaller subset of orientations.^[^ ^15^, ^35^ ^]^ While straightforward, this geometric assessment does not explain the entirety of the κ_21_ growth dependence. The asymmetric tendency for larger κ_21_ when growing *toward* the <100> direction indicates there must be corresponding changes in the dislocation density with lattice orientation.

**Figure 2:**
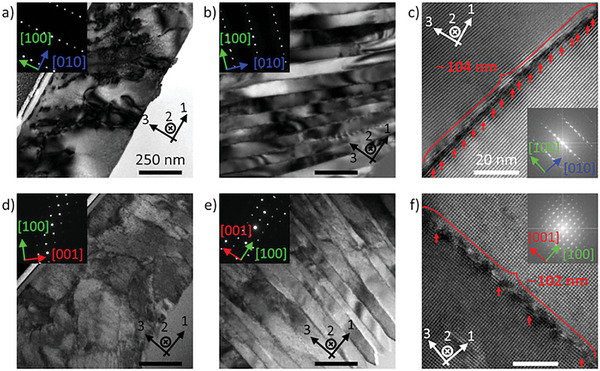
Bright‐field and high‐resolution TEM images of Sb_2_S_3_ crystals grown in stoichiometric glass near orientations of interest indicated by inset selected area electron diffraction patterns or fast‐Fourier transforms. Top row: crystal fabricated at 3.5 mW laser power and 100 µm s^−1^ scanning speed rotating about the <001> crystallographic axis. Bottom row: same as top row, but for 5 mW, 25 µm s^−1^, and rotation about <010>. First column: growth nearly perpendicular to the <100> axis with lower and diffuse dislocation densities. Second column: growth nearly parallel to the <100> axis with higher dislocation densities aligned into series of low‐angle grain boundaries forming lamellae. Last column: magnified images of grain boundaries as collections of aligned ½[100] dislocations with differing dislocation cores. Black and white scale bars indicate 250 and 20 nm respectively.

To determine changes in dislocation density, transmission electron microscopy (TEM) samples were prepared as longitudinal cross sections (the plane defined by the 1‐ and 3‐axes) for growth directions near the principal crystallographic axes. The first row of micrographs in **Figure** **2** shows a sample prepared with slightly higher laser power (3.5 mW) compared to Figure 1. This crystal rotates nearly parallel to the <001> crystal axis with growth directions nearly parallel to the <010> and <100> crystal axes for Figure 2a,b, respectively. Along this crystal, dislocation cores were nearly parallel to the axis of rotation (2‐axis) and the local strain fields around each dislocation acted to arrange themselves relative to one another according to the overall dislocation density. Growth near the <010> crystal axis shows only partial dislocation arrangement whereas growth near the <100> crystal axis has a much higher relative dislocation density as evidenced by the full alignment of dislocations into a series of low‐angle tilt boundaries forming lamellae 50–100 nm in width; this arrangement was not observed in previous rotating lattice Sb_2_S_3_ crystals.^[^ ^32^ ^]^ Although not directly comparable, these general trends were repeated for another crystal cross section grown with different laser parameters and rotating nearly about the <010> crystal axis as shown in the bottom row of Figure 2. For this other axis of rotation, growth nearly parallel to the <100> axis in Figure 2e shows low‐angle tilt boundaries and lamellae formation from higher dislocation densities compared to the partial dislocation arrangement of growth perpendicular to the <100> axis and nearly parallel to the <001> axis – Figure 2d. Significant lattice rotation about the <100> crystal axis was not observed for any crystals grown in the present work implying this lattice orientation is either unstable or disfavored by biased seed orientations.^[^ ^34^ ^]^

Higher magnification lattice images were acquired to determine the misorientation across the low‐angle tilt boundaries observed in Figure 2b,e. The tilt‐boundary misorientation angle can be measured directly from the fast‐Fourier transform or calculated from the linear density of dislocation Burgers vectors.^[^ ^36^ ^]^ Figure 2c shows a series of 1/2[100] dislocations imaged along their <001> dislocation cores with a calculated misorientation angle of 5.61° closely matching the measured angle of 5.67°. Figure 2f shows a similar result for a lower dislocation density tilt‐boundary also composed of 1/2[100] dislocations, but with <010> cores, and calculated and measured misorientation angles of 1.27° and 1.30°, respectively. Overall, the tilt boundary misorientation angles ranged from 1 to 6° independent of the type of dislocation core. Furthermore, the surface normal of the low‐angle tilt boundaries was always parallel to the 1/2[100] Burgers vector and rotates along with the lattice as the crystal grows through the <100> direction. Based on the tilt‐boundary misorientation angle and lamellae size, the theoretical maximum value for κ_21_ could be well in excess of 10° µm^−1^, but this value is never directly measured by EBSD due to the > 100 nm step size used for acquiring the datasets and the processing restrictions for approximating the lattice curvature components.

Regardless of the exact dislocation arrangement observed in Figure 2, the dislocation density tends to increase for crystals growing closer to the <100> crystal axis independent of the axis of rotation. The increased dislocation densities – at least for the predominant 1/2[100] dislocations – implies the dislocations become more energetically favorable for these same growth directions. Although susceptible to alignment imperfections during sample preparation, the crystal thickness in the TEM samples also increased for crystals growing near <100> axis from ≈0.5 µm elsewhere to ≈1 µm. A corresponding volume increase and associated increase in local densification stresses may drive the greater dislocation densities, but the anisotropy of Sb_2_S_3_ crystal growth rates and elastic moduli may also contribute. Alternatively, Stukenberg et al.^[^ ^37^, ^38^ ^]^ proposed that existing dislocations could serve as nucleation sites for further dislocation formation via an autodeformation mechanism, which could propagate higher dislocation densities at grain boundaries of certain orientations. Overall, a combination of changes in the Burgers vector projection along the crystal growth direction and relative changes in the dislocation density impart a crystallographic dependence on the predominant κ_21_ lattice curvature, both of which maximize κ_21_ as it grows toward and through the <100> crystal direction.

#### 2.1.2 Formation of Secondary Lattice Curvatures

**Figure 3:**
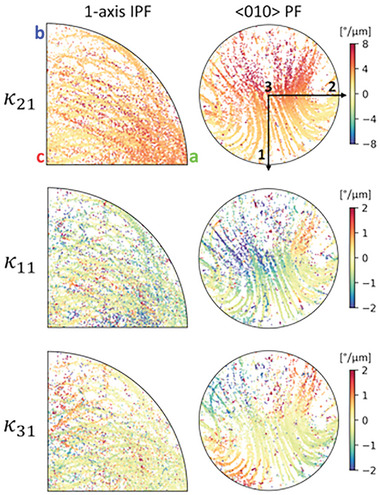
Aggregate orientations and κ_*i*1_ lattice curvature component data for 30 Sb_2_S_3_ crystal lines ≈80 µm long fabricated in stoichiometric glass with the same laser parameters as Figure 1. κ_*i*1_ data is plotted as a 1‐axis IPF unit triangle and a <010> axis PF. κ_21_ is maximized when growing toward the <100> crystal direction and the other κ_*i*1_ components are about four times smaller and are antisymmetric about certain orientations.

Local lattice curvature determined from EBSD datasets was resolved into the first six accessible tensor components (κ_*i*1_ and κ_*i*2_). Of these components, only κ_*i*1_ can significantly contribute to the evolution of lattice orientation along a crystal line, whereas the κ_*i*2_ components affect the transverse crystal orientation (which may be significant for planar architectures^[^ ^39^ ^]^). For the rotating lattices investigated in this work, the predominant lattice curvature component was always κ_21_, but significant rotations about the 1‐ and 3‐axes along the crystal length (κ_11_ and κ_31_) were also observed. **Figure** **3** shows the same aggregated data for the 30 crystal lines used to generate the bottom 1‐axis IPF in Figure 1, but for all three κ_*i*1_ components plotted in their 1‐axis IPF unit triangle and <010> axis pole figure (PF) – the other stereographic projections are provided in Figures S2 and S3 (Supporting Information). The magnitude of the secondary lattice curvature components is about four times smaller (< 2° µm^−1^) than κ_21_, and includes both positive and negative values (i.e., left‐ and right‐handed rotations). Focusing on the <010> PFs, the secondary lattice curvature components have regions of lattice orientations that are entirely positive or negative, which appear antisymmetric about certain orientations. For example, κ_31_ is positive with the crystal growing to one side of the <010> axis but is negative when growing along the other side; this behavior is somewhat reversed for κ_11_. The antisymmetry of the secondary lattice curvature components implies that there are stable (or metastable) lattice orientations where κ_21_ is positive, but the other κ_*i*1_ components vanish. This behavior would tend to align the lattice to rotate about certain crystallographic axes, but the ≈80 µm long crystals lines in this particular experiment do not allow enough total lattice rotation to unambiguously observe such behavior. Furthermore, κ_21_ alone cannot reorient the axis of rotation, so any alignment must be entirely due to the presence of these secondary lattice curvature components.

The mechanism forming the secondary lattice curvature components must either form plastically from the dislocation structure driving κ_21_ (see Figure 2) or elastically from strain gradients according to Equation (2). Considering only plastic contributions, dislocation densities are physically bounded such that ρ_*j*_ ≥ 0. Figure 3 shows the measured κ_21_ is always positive; thus, *b*_1_ ≥ 0 for all lattice orientations following predominantly plastic deformation, where κ_21_ = *b*_1_ρ_2_. The plastic contributions of secondary lattice curvatures from Equation (2) are κ_31_ = *b*_1_ρ_3_ and $\kappa_{11}=1/2\left(b_{1}\rho_{1}-\;b_{2}\rho_{2}-b_{3}\rho_{3}\right)$. Under these restrictions, purely plastic κ_31_ must also always be positive, which Figure 3 disproves, necessitating the contribution of elastic strain gradients to explain, at least part of, the secondary lattice curvature components. Although it appears possible from the TEM analysis, these strain gradients have yet to be directly measured since the TEM sample preparation method cannot guarantee that elastic strains will remain unaltered.

### 2.2 Extended Crystal Growth

#### 2.2.1 Formation of Macroperiodic Structures

**Figure 4:**
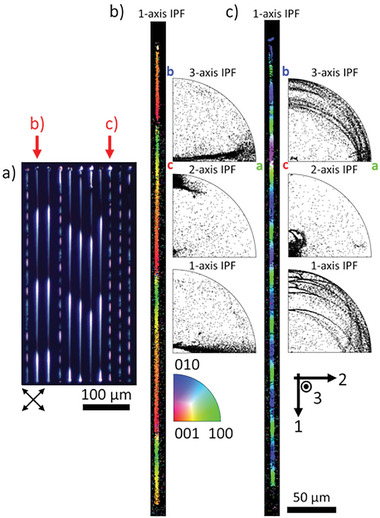
Macroperiodic Sb_2_S_3_ rotating lattice crystals fabricated in stoichiometric glass with 3.5 mW laser power scanned at 100 µm s^−1^. a) POM image of crystal lines showing two distinct macroperiods. EBSD of selected lines with 1‐axis IPF colored maps and IPFs of each major sample axis for b) long and c) short macroperiodicity. 2‐axis IPFs indicate the predominant axis or rotation of <010> and <001> crystal axes for long and short macroperiodicities, respectively.

Investigations of initial crystal growth show the presence of secondary lattice curvature components antisymmetric about certain lattice orientations. Due to the smaller scale of secondary lattice curvature components (< 2° µm^−1^), observing alignment effects required fabrication of much longer crystal lines (≈450 µm or more) than previously reported.^[^ ^15^ ^]^ **Figure** **4**a shows a polarized optical microscope (POM) image of a collection of longer Sb_2_S_3_ crystal lines fabricated in stoichiometric glass with 3.5 mW laser power scanned at 100 µm s^−1^. The most striking feature of these lines is an unexpected periodic change in color along their lengths indicating periodic changes in lattice orientation confirmed by EBSD. For a 45° rotation of the polarizer and analyzer as shown here, Figure 4b shows the white and dark colors indicate the <100> and <001> crystal axes oriented vertically, respectively, with the <010> crystal axis oriented horizontally. Similarly, Figure 4c shows the red and blue colors indicate the <100> and <010> crystal axes oriented vertically, respectively, with the <001> crystal axis oriented horizontally. In order to distinguish between these microscale periodic changes in the rotating lattice orientation from the nanoscale translational periodicity of the unit cell, we refer to this much larger, new periodicity as macroperiodicity. Unlike examples of bending lattice curvature,^[^ ^1^, ^24^, ^25^ ^]^ lattice curvature persists in these rotating lattice crystal lines through several full macroperiods further indicating this lattice curvature is intrinsic to the crystal growth process.

Two distinct macroperiodic structures are observed in Figure 4a with average repeat units of 153 ± 6 µm and 50 ± 5 µm. Figure 4b,c are EBSD scans of each type of line with the longer, 152 ± 3 µm; and shorter, 48 ± 3 µm, macroperiods. Plotted next to each 1‐axis IPF colored maps are the 1‐, 2‐, and 3‐ axis IPF unit triangles. The 2‐axis IPF shows a clustering of lattice orientations indicating alignment about a common crystallographic axis of rotation along each line. The longer macroperiodicity rotates about the <010> crystal axis and the shorter macroperiodicity about the <001> crystal axis; stable rotation was not observed about the <100> crystal axis. The macroperiod length is inversely proportional to the average lattice curvature, therefore rotation about the <001> axis generally has greater lattice curvature,≈3.8° µm^−1^, than the <010> axis rotation with 1.7° µm^−1^. The macroperiod length and lattice curvature magnitude depend on the laser parameters following previously observed trends,^[^ ^1^ ^]^ where increasing laser power or laser scanning speed decreases the average lattice curvature magnitude and increases the macroperiod length. The axis of rotation is further biased by these laser parameters, favoring rotation about the <010> crystal axis with increasing laser power and laser scanning speed. A detailed investigation into the correlation between laser parameters and macroperiod lengths is underway and will be presented in a future publication.

#### 2.2.2 Evolution of Lattice Alignment

**Figure 5:**
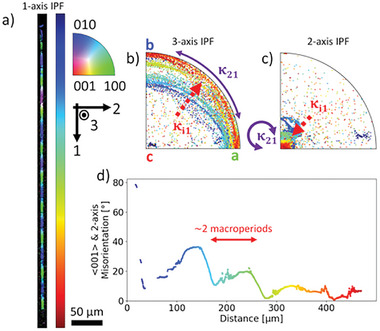
Alignment of crystal orientation from the crystal in Figure 4c. a) 1‐axis IPF colored map with color bar indicating length along the crystal line. b) 3‐axis and c) 2‐axis IPFs with lattice curvature components indicated. κ_21_ lattice curvature persists throughout the alignment. d) Plot of <001> and 2‐axis misorientation along crystal length showing non‐monotonic alignment.

Due to the constant laser scanning speed, the length along each crystal line acts as a pseudo‐record of the lattice orientation as the crystal is grown. **Figure** **5** shows this alignment process for the shorter macroperiodicity or rotation about the <001> crystal axis for the same crystal line as in Figure 4c – the crystal line in Figure 4b is nearly aligned at the start. To aid the static analysis in Figure 5 animated figures for each line are provided as Figure S4 and S5 (Supporting Information). Figure 5b,c are the 3‐ and 2‐axis colored IPFs according to the distance along the crystal lines shown in Figure 5a. Next to each IPF is the predominant κ_21_ lattice curvature component driving lattice rotation and the other secondary κ_*i*1_ lattice curvature components. Figure 5d shows the misorientation between the <001> crystal axis and axis of rotation (2‐axis) along the line. The lattice is initially misaligned (> 70°) but it quickly approaches better alignment, likely due to some grain boundary formation immediately following the seed crystal.^[^ ^33^ ^]^ After the seed, the <001> and 2‐axis are more closely aligned (< 30°), which progressively and non‐monotonically improves along the length of the line. Notably, κ_21_ alone is insufficient to produce the type of alignment observed in Figure 4, necessitating the effect of the secondary lattice curvature components, κ_*i*1_. Furthermore, the gradual but persistent alignment about the <001> axis indicates this phenomenon is not happenstance, but instead some active process during crystal growth.

The non‐monotonic alignment of the crystal lattice shown in Figure 5d may be better described as the lattice ‘wobbling’ into a more stable rotational configuration. This behavior occurs across two macroperiod lengths where the crystal initially aligns toward the <001> axis of rotation for the first macroperiod, then misaligns to a lesser degree across the next macroperiod. This behavior can be explained by the axial mirror symmetry of the Sb_2_S_3_ point group ($\frac{2}{m}\frac{2}{m}\frac{2}{m}$). Consider a simplified case of a misaligned lattice orientation with only κ_21_ lattice curvature – no alignment. If we track some axis (e.g., the <010>), the crystal at some point along a macroperiod will be growing to the “right” of this axis. After 180° of rotation or one macroperiod, the crystal will now be growing to the “left” of this axis, which then repeats. The secondary lattice curvature components in Figure 3, κ_11_ and κ_31_, also change signs when growing to either side of certain lattice orientations (e.g., <010> in this example). For one macroperiod the sum contribution of the secondary lattice curvature components then acts to align the axis rotation, but the next macroperiod may differ, repeating only every 360° of rotation, or two macroperiods. These slight differences are not clear in the POM images like in Figure 4a but can be seen as slight differences in color in the 1‐axis IPF colored maps from EBSD analysis. Overall, the crystal orientation does not repeat until a full 360° rotation, except when the crystal is fully aligned about the <010> or <001> axis where the point group symmetry reduces this to every 180° of rotation.

#### 2.2.3 Mechanism of Lattice Alignment

Lattice alignment toward rotation about either the <010> or <001> crystal axes can be considered with respect to the predominant 1/2[100] dislocations governing lattice curvature in Sb_2_S_3_^[^ ^40^ ^]^ and Equation (2). These two axes correspond to the principal dislocation core directions for 1/2[100] edge dislocations, and further lack of stable rotation about the <100> crystal axis implies this behavior is not coincidental. Considering a generally misaligned lattice as expected from a stochastic or nearly stochastic^[^ ^34^ ^]^ seed orientation, κ_*i*1_ will be unbounded so long as κ_21_ is positive – as is the case measured in Figure 3. In contrast for a perfectly aligned lattice, the predominant 1/2[100] dislocation cores will align parallel to the 2‐axis, or the dislocation density will be present entirely as ρ_2_, while ρ_3_ ≈ ρ_1_ ≈ 0. The Burgers vector will be further confined entirely within the plane defined by the 1‐ and 3‐axes, or *b*_2_ ≈ 0. The predominant mechanism for plastic lattice curvature, the 1/2[100] dislocations, can then only contribute toward κ_21_ lattice curvature, and any mechanism to destabilize this rotating configuration as secondary lattice curvature components must be elastic or come from the two dislocations with smaller Burgers vectors. Even for just the predominant κ_21_ lattice curvature component, a single slip system is insufficient to explain full macroperiodic lattice rotation. The smaller 1/4[102], and $1/4[\bar{1}02]$ edge dislocations will have some contribution^[^ ^32^ ^]^ and for <001> crystal axis growth are the only plastic contributions toward nonzero κ_21_. Elastic strain gradients may also generally affect the overall lattice curvature. Specifically for the case of <010> crystal axis growth, *b*_1_ vanishes for all known slips systems, yet κ_21_ is still measured as nonzero, necessitating elastic contributions toward κ_21_ lattice curvature as well. Overall, while 1/2[100] dislocations govern the majority of Sb_2_S_3_ lattice curvature, this slip system alone is insufficient to provide a full description.

**Figure 6:**
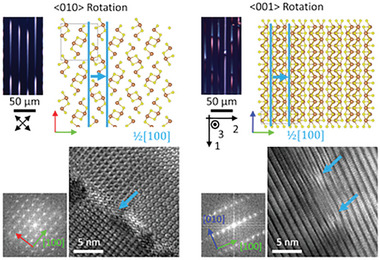
Predominant 1/2[100] dislocation cores for (left) <010> and (right) <001> axes of rotation. (Top) POM and extended crystal structures along the axis of rotation with 1/2[100] Burgers vectors indicated. (Bottom) high‐resolution TEM of 1/2[100] dislocations (indicated with arrows) and respective fast‐Fourier transforms with colored arrows showing principal crystallographic directions.

Alignment toward either of the preferred axes of rotation can also be considered with regards to the 1/2[100] dislocations. Despite the same Burgers vector length, the two dislocation cores do not form the same type of half‐planes within the lattice. **Figure** **6** shows a comparison of the two dislocation core directions including POM images of their respective crystal lines, high‐resolution TEM images of dislocation cores after several macroperiods of lattice alignment, and similarly oriented extended models of the Sb_2_S_3_ crystal lattice with the 1/2[100] Burgers vector indicated by the light blue arrows.^[^ ^41^ ^]^ Formation of <010> dislocation cores generate half‐planes between the [Sb_4_S_6_]_n_ chains whereas formation of <001> dislocation cores requires half‐plane formation across these same chains, likely requiring more energy. Changes in relative dislocation formation energies may bias toward one dislocation core direction over another subsequently biasing the lattice toward rotation about a particular crystal axis, which may then be biased by the laser power or laser scanning speed. Despite these factors, the lattice orientation of the initial seed propagates along the crystal line, influencing the preferred axis of rotation. The initial lattice orientation is usually considered stochastic, but Au‐Yeung et al.^[^ ^34^ ^]^ have demonstrated the glass surface and laser polarization may offer methods to bias the initial lattice orientation and by extension the final axis of rotation.

## 3 Conclusion

By extending the length of rotating lattice single crystal lines in glass to several hundred micrometers, a new macroperiodicity has been discovered, providing a basis of novel metastructures in the Sb_2_S_3_ system. Understanding the locally changing crystallographic dependence of these lattices subjected to crystallization stresses is important for the practical utilization of these unique metastructures. Crystals with lower symmetry and limited slips systems lead to anisotropic lattice curvature. Misaligned dislocations and elastic contributions further give rise to smaller secondary lattice curvatures and potential alignment effects over extended crystal growth, forming larger (10s of microns) macroperiodic metastructures. Despite these crystallographic effects, stresses from crystal fabrication are continuously generated driving intrinsic lattice curvature, regardless of the efficiency of the available deformation mechanisms to relieve these stresses.

Specifically, for model rotating lattice of Sb_2_S_3_ crystal lines, many of the observed phenomena are governed by the predominant 1/2[100] dislocation mediating lattice curvature. The magnitude of the predominant lattice curvature component (κ_21_), strongly depends on the crystal growth direction, maximizing when growing toward and through the <100> crystal growth direction due to alignment of the Burgers vector and corresponding increases in dislocation density. Higher dislocation density leads to alignment of dislocation cores into a series of low‐angle (1 – 6°) tilt‐boundaries forming lamellae 50–100 nm in width. Furthermore, a combination of misaligned Burgers vectors, dislocation cores, and elastic strain gradients develop into secondary lattice curvature components, κ_*i*1_, about four times smaller than κ_21_, which are antisymmetric about certain lattice orientations.

The antisymmetry of the secondary lattice curvature components aligns the crystal lattice to rotate about either the <001> or <010> crystal axis. Predominant κ_21_ persists throughout this alignment still varying with growth direction, eventually forming repeating structures of lattice orientation. These repeating macroperiodic metastructures range from 20 to 160 µm in length depending on the laser parameters and axis of rotation. Rotation about the <001> axis forms shorter macroperiods, or larger average lattice curvatures, which is reversed for <010> crystal axis rotation. These axes of rotation coincide with the principal dislocation core directions of the 1/2[100] dislocation, where no stable rotation was observed about the <100> crystal axis.

## 4 Experimental Section

### Common Description of Lattice Curvature

Lattice curvature can be described by a second‐rank tensor defined as the slope of an axial rotation vector, ω_*i*_, over a direction, *x_j_*. Equation (1) defines the lattice curvature tensor, κ_*ij*_, which is often approximated experimentally.

(1) $$\kappa_{ij}=\frac{\partial \omega_{i}}{\partial x_{j}}\approx \frac{\Delta \omega_{i}}{\Delta x_{j}}$$

Plastic contributions toward lattice curvature were formalized by Nye^[^ ^19^ ^]^ as a geometrically necessary dislocation density, α_*ij*_, which itself is the product of the i^th^ component of a dislocation's Burgers vector, *b_i_*, and the dislocation density through a surface, ρ_*j*_, or α_*ij*_ = *b_i_* ρ_*j*_. This relation was expanded by Kröner^[^ ^20^ ^]^ to include elastic contributions from strain, ε_*ij*_, gradients along a direction, *x_k_*. The total plastic and elastic contributions of lattice curvature are accounted for in Equation (2) where δ_*ij*_ is the Kronecker delta and ε_*ikl*_ is the permutation operator.

(2)

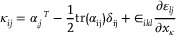




To effectively use the lattice curvature tensor, the common coordinate system was used for laser crystallization of glass introduced by Musterman et al.^[^ ^1^ ^]^ In this system the 1‐axis is defined by the laser scanning direction and predominant crystal growth direction. The 3‐axis is opposite the laser propagation direction and normal to the sample surface. Finally, the 2‐axis is perpendicular to the 1‐ and 3‐axes to form a right‐handed coordinate system. Within this system, the rotation rates previously reported for rotating lattice single crystal lines are defined entirely by one nonzero and positive κ_21_ component.^[^ ^15^ ^]^ Similarly, spiral crystal growth would be only κ_11_^[^ ^16^, ^17^ ^]^ and graded lattices would be a combination of κ_23_ and κ_32_.^[^ ^18^ ^]^ This coordinate system has been indicated within all figures used throughout this work.

### Glass Sample Preparation

All crystals fabricated in this work were grown in stoichiometric Sb_2_S_3_ glasses prepared by the ampule quench method following Savytskii et al.^[^ ^15^ ^]^ Sb_2_S_3_ powders (Alfa Aesar, 99.999%) were flame sealed under vacuum (10^−2^ torr) in 1 mm diameter fused quartz ampules with 10 µm wall thickness. These ampules were then heated to 650 °C at 2 °C min^−1^ in a custom‐built rocking furnace and held for ≥ 10 h at the maximum temperature before quenching in water. After quenching, glass pieces were mounted and polished to optical quality finishing with 50 nm colloidal silica, and checked with a POM (Olympus BH2‐UMA) to ensure the entire surface was amorphous.

### Laser Crystallization

Laser crystallization for stoichiometric Sb_2_S_3_ glass was achieved on the glass surface using a 639 nm continuous wave diode laser (ThorLabs LP637‐SF70). Collimated light was focused through an objective lens (Olympus MPlan 50X, 0.75NA) ≈2 µm above the glass surface to provide a divergent spot size ≈7 µm in diameter. Specific laser powers and scanning speed are reported for each specific experiment. Laser polarization was aligned along the laser scanning direction using a liquid‐crystal‐on‐silicon spatial light modulator (Hamamatsu LCOS‐SLM, X10468 Series) following Au‐Yeung et al.^[^ ^34^ ^]^; this component acted simply as a polarizing mirror for all experiments in this work. Laser crystallization occurred with the beam perpendicular to the surface and under flowing nitrogen gas to prevent significant oxidation.

All glasses used in this work readily crystallize under laser irradiation to the orthorhombic Sb_2_S_3_ stibnite phase (*Pnma*, *a* = 11.314 Å, *b* = 3.837 Å, *c* = 11.234 Å).^[^ ^42^ ^]^ This crystal is comprised of [Sb_4_S_6_]_n_ chains extending infinitely along the *b*‐axis which also constitutes the fast crystal growth direction.^[^ ^43^ ^]^ Perpendicular to these chains within the (010) plane, Sokol et al.^[^ ^40^ ^]^ measured the preferred slip systems as three edge dislocations: 1/2[100], 1/4[102], and $1/4[\bar{1}02]$ with Burgers vector lengths of 5.66, 1.26, and 1.26 Å, respectively.

### Optical and Electron Microscopy

Reflection POM was used to quickly distinguish the isotropic glass from the biaxial birefringent Sb_2_S_3_ crystal^[^ ^44^ ^]^ and qualitatively distinguish crystal orientation. Changes in reflected color depend on the illumination source, light interaction depth, and the local birefringence magnitude, the latter of which is strongly correlated with lattice orientation. POM images were acquired with an Olympus BH2‐UMA optical microscope using reflected incandescent illumination. Polarizer and analyzer directions are indicated for all POM images.

Three electron microscopes at the Materials Characterization Facility of the Institute for Functional Materials and Devices at Lehigh University were used in this work. Topographic images and EBSD datasets were acquired using a Hitachi 4300 SE variable pressure SEM. Samples were uncoated, and experiments were performed with 30 Pa of ambient air to prevent significant sample charging. EBSD patterns were acquired with a Hikari camera using EDAX TSL OIM data acquisition software at 30 kV accelerating voltage and the conventional 70° sample tilt. Pattern orientations were indexed to an Sb_2_S_3_ crystal reference and were assigned several quality parameters (image quality, confidence index, and fit). These quality parameters were used to distinguish between glass and crystal, and to discount misindexed pixels from further analysis.

A JEOL JEM 2100 TEM operated with an accelerating voltage of 200 kV was used for higher magnification and lattice imaging, and dislocation analysis. No charging was observed during TEM characterization. Electron transparent samples were prepared for the TEM using an FEI Scios dual‐beam SEM equipped with a focused ion beam (FIB) following the FIB milling procedure used by Musterman et al.^[^ ^32^ ^]^ Samples were coated with 1 nm of Ir and electrically grounded to prevent charging.

### Determination of Local Lattice Curvature

In previous studies,^[^ ^15^, ^32^ ^]^ the rotation rates (κ_21_) of Sb_2_S_3_ rotating lattice single crystals were determined by fitting the gradient of misorientation across full crystal lines. This averaged approach works well for small misorientations that are predominantly rotating about a single axis but cannot capture changes in lattice curvature within the integration area or rotation about additional sample axes. In this study, a procedure was developed modified from Pantleon and He et al.^[^ ^45^, ^46^ ^]^ to determine lattice curvature on a pixel‐by‐pixel basis from 2D EBSD datasets. For every pixel in the EBSD dataset, pairwise misorientations were determined relative to each neighboring pixel within some distance (e.g., 2.5 µm). These misorientations were then decomposed into rotations about the three sample axes, and their gradients to each mapping direction were determined via linear least‐squares regression. The resulting slopes are approximations of the first six lattice curvature components with the 95% confidence interval for each slope typically <1% of the total value. To avoid contributions from the glass and potentially misindexed pixels, datasets were filtered by the EBSD quality parameters (normalized image quality > 15–60%, confidence index > 0–0.1, and fit‐factor < 1.9–2.2°) and any individual misorientations above some cut‐off threshold depending on the window size (κ_*ij*_ > 10° µm^−1^ for this work). A Python implementation of this procedure can be found at ref. [47].

## Conflict of Interest

The authors declare no conflict of interest.

## Supporting information



Supporting Information

Supporting Figure 4

Supporting Figure 5

## Data Availability

Data underlying the results presented in this paper are not publicly available at this time but may be obtained from the authors upon reasonable request.
