# Supplementary material for: Dislocation‐Driven Formation of Oriented Macroperiodic Metastructures of Curved Single Crystal Lattices in Glass
Source: Adv Sci (Weinh). 2025 Feb 4;12(12):2412833. doi: 10.1002/advs.202412833 (PMC11947992; doi:10.1002/advs.202412833)
Supplement: Supplementary file 1 — Supporting Information [file ADVS-12-2412833-s002.docx]

# SUPPORTING INFORMATION

**Dislocation-driven formation of oriented macroperiodic metastructures of curved single crystal lattices in glass**

Evan J. Musterman, Volkmar Dierolf, Himanshu Jain^*^

## Full Description of $\kappa_{i1}$ Lattice Curvature

The lattice orientations for the 30 crystal lines used to create the bottom inverse pole figures (IPFs) in Figure 1 and Figure 3 can be viewed along different stereographic projections for different perspectives of the crystallographic dependence of the $\kappa_{i1}$ lattice curvature components. For the sake of completeness, the three IPF unit triangles about each sample axis, and pole figures (PFs) about each crystallographic axis are included for $\kappa_{21}$ in Figure S1, $\kappa_{11}$ in Figure S2, and $\kappa_{31}$ in Figure S3. All of the information is the same as in the main text, just viewed along different axes.


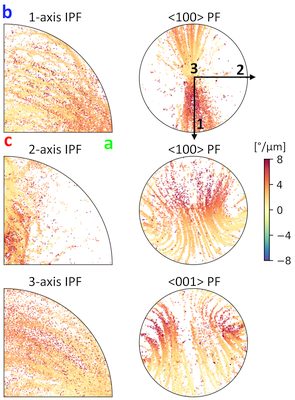


Figure S1: Aggregate orientations and $\kappa_{21}$ data for 30 Sb_2_S_3_ crystal lines ~80 µm long fabricated in stoichiometric glass with the same laser parameters as Figure 3. Data is displayed as IPF unit triangles about each sample axis and PFs for each crystallographic axis.


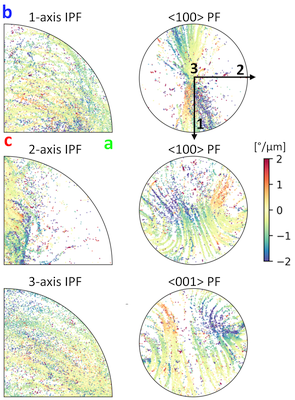


Figure S2: Aggregate orientations and $\kappa_{11}$ data for 30 Sb_2_S_3_ crystal lines ~80 µm long fabricated in stoichiometric glass with the same laser parameters as Figure 3. Data is displayed as IPF unit triangles about each sample axis and PFs for each crystallographic axis.

## Evolution of Lattice Alignment

Alignment of the crystal lines highlighted in Figure 4 may be more readily understood in an animated format. Figure S4 shows the evolution of lattice orientation along the crystal line in Figure 4b for rotation about the <010> crystal axis or the longer macroperiodicity. This alignment is shown for the three IPF unit triangles along the sample axes and the three PFs about each crystallographic axis for completeness. Figure S5 is a similar representation of the crystal line in Figure 4c for rotation about the <001> crystal axis or the shorter macroperiodicity. The crystal lines in Figure 4b and S4 are more closely aligned than Figure 4c and S5, so any alignment is less pronounced along the crystal length.


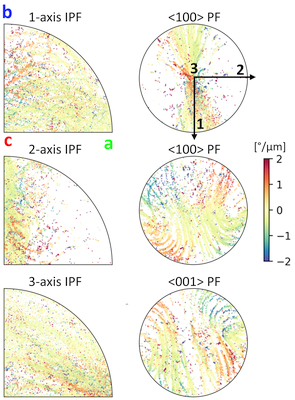


Figure S3: Aggregate orientations and $\kappa_{31}$ data for 30 Sb_2_S_3_ crystal lines ~80 µm long fabricated in stoichiometric glass with the same laser parameters as Figure 3. Data is displayed as IPF unit triangles about each sample axis and PFs for each crystallographic axis.

Figure S5: Animated evolution of the lattice of the crystal line in Figure 4c with the shorter macroperiodicity and rotation about the <001> crystal axis. Left is the 1-axis IPF colored map with colorbar indicating length along the crystal line. Right are the IPF unit triangles about each sample axis and PFs about each crystallographic axis.

Figure S4: Animated evolution of the lattice of the crystal line in Figure 4b with the longer macroperiodicity and rotation about the <010> crystal axis. Left is the 1-axis IPF colored map with colorbar indicating length along the crystal line. Right are the IPF unit triangles about each sample axis and PFs about each crystallographic axis.
